# Sodium new houttuyfonate suppresses metastasis in NSCLC cells through the Linc00668/miR-147a/slug axis

**DOI:** 10.1186/s13046-019-1152-9

**Published:** 2019-04-11

**Authors:** Rilei Jiang, Cheng Hu, Qian Li, Ziyu Cheng, Ling Gu, Hongxiao Li, Yuanyuan Guo, Qirui Li, Yueyang Lu, Ke Li, Meijuan Chen, Xu Zhang

**Affiliations:** 1 School of Medicine and Life Sciences, Nanjing University of Chinese Medicine, Nanjing, 210023 People’s Republic of China; 2 Institute of Literature in Chinese Medicine, Nanjing University of Chinese Medicine, Nanjing, 210023 People’s Republic of China; 3 School of Medicine and Life Sciences and Jiangsu Collaborative Innovation Center of Traditional Chinese Medicine (TCM) Prevention and Treatment of Tumor, Nanjing University of Chinese Medicine, Nanjing, 210023 People’s Republic of China

**Keywords:** Sodium new houttuyfonate (SNH), Linc00668, miR-147a, NSCLC, Metastasis, ceRNA

## Abstract

**Background:**

As most lung cancer patients present with invasive, metastatic disease, it is vital to investigate anti-metastatic treatments for non-small cell lung cancer (NSCLC). *Houttuynia cordata* is commonly used as a Chinese anticancer medicine in the clinic, and sodium new houttuyfonate (SNH), a main compound of this herb, has long been found to have antibiotic effects, although its anticancer effects have not been investigated. Here, we tried to address this lack of research from the perspective of the competing endogenous RNA (ceRNA) theory.

**Methods:**

The effects of SNH on NSCLC cells were analysed with Cell Counting Kit-8 assays and colony formation assays. In addition, transwell assays and wound healing assays were used to determine the effects of SNH on migration and invasion in NSCLC cells. The levels of key genes and proteins were examined by quantitative real-time PCR, western blotting, immunofluorescence staining and IHC staining. Through transcriptome screening and digital gene expression profiling, Linc00668 was identified to be regulated by SNH. Dual-luciferase reporter assays and RNA immunoprecipitation assays verified the binding efficiency between miR-147a and Linc00668 or Slug.

**Results:**

In the present study, SNH regulated NSCLC cells in multiple ways, the most prominent of which was suppressing the expression of Linc00668, which was indicated to promote migration and invasion in NSCLC cells. Functional studies demonstrated that Linc00668 acted as a ceRNA by sponging miR-147a to further regulate Slug mRNA levels, thereby influencing the progression of the epithelial-mesenchymal transition. Consistently, the results of in vivo animal models showed that SNH depressed Linc00668 and suppressed the metastasis of NSCLC.

**Conclusions:**

SNH suppressed metastasis of NSCLC cells and the mechanism may involve with the Linc00668/miR-147a/Slug axis.

**Electronic supplementary material:**

The online version of this article (10.1186/s13046-019-1152-9) contains supplementary material, which is available to authorized users.

## Background

Lung cancer is the most common cause of cancer death and was responsible for one-quarter of cancer deaths last year [1]. Despite improvements in the development of targeted drugs and advances in treatment measures, the overall 5-year survival rate for lung carcinoma is still less than 15% due to limited therapeutic options, tumour metastasis, and recurrence [2]. Given the vicious biological behaviour of lung cancer, it is vital to seek out additional measures to control the disease.

Metastasis, which is responsible for 90% of cancer deaths worldwide, is one of the primary characteristics of lung cancer [3]. During metastasis, epithelial tumour cells invade the nearby extracellular matrix (ECM), expand into the systemic circulation and contribute to secondary tumours at distant sites [4]. The epithelial-mesenchymal transition (EMT) is a process in which stationary epithelial cells lose their epithelial characteristics and gain a mesenchymal morphology with the ability to migrate and invade [5]. Several EMT drivers, such as Snail, Slug and TCF8/ZEB1, have been closely associated with recurrence and survival in patients with breast, colorectal and ovarian cancer, indicating that the EMT process leads to poor clinical outcomes [6].

Natural products (NPs) have been used as traditional medicines since antiquity [7]. Elucidating the mechanisms of the compounds from traditional medicine is important to support clinical usage and inspire treatment strategies. *Houttuynia cordata* Thunb. is a traditional Chinese herb that has been used to treat lung diseases for thousands of years. Researches of *Houttuynia cordata* Thunb on lung cancer is obviously. Han K et al. declared that *Houttuynia cordata* is of potential value in the treatment of lung cancer, although the underlying mechanisms need to be further confirmed [8]. The main ingredient of *Houttuynia cordata*, sodium houttuyfonate (SH), has been used for the treatment of purulent skin infections and respiratory tract infections [9]. Because of the chemical instability of houttuynin, its addition product, sodium new houttuyfonate (SNH), has been synthesized for improved stability. Previous studies have indicated that SNH/SH can significantly repress various kinds of bacteria, including *Staphylococcus aureus*, *Bacillus subtilis*, and *Pseudomonas aeruginosa* [10–12]. Recent studies have even revealed that SNH inhibits the inflammatory response through NF-κB-associated signalling pathways such as the TLR4/NF-κB and MAPKs/NF-κB pathways [13, 14]. However, although *Houttuynia cordata* Thunb. is frequently used to treat lung cancer in Chinese clinics, there have been no further in-depth studies on its mechanisms.

A large proportion of the human genome is transcribed as noncoding RNAs (ncRNAs) [15]. Long ncRNAs (lncRNAs) demonstrate multiple functions, including nuclear sequestration, modulation of chromosomal interactions, chromatin looping, gene methylation and chromatin modification, in various malignant tumours such as lung adenocarcinoma, breast carcinoma, gastric cancer and hepatocellular carcinoma [16–19]. Among the potential mechanisms, the competing endogenous RNA (ceRNA) theory has received much recognition based on mounting evidence [20]. In the ceRNA theory, lncRNAs communicate or co-regulate by competing with or binding with shared microRNAs, which are small ncRNAs that play important roles in the post-transcriptional regulation [21].

In this study, we first confirmed that SNH could restrain NSCLC progression in multiple ways, especially by regulating migration and invasion. Then, we attempted to explain the mechanism with ceRNA theory. We found that Linc00668 was suppressed dramatically by SNH treatment in NSCLC cells, and upon further investigation, a Linc00668/miR-147a/Slug axis was discovered that could markedly modulate migration, invasion and the EMT in NSCLC cells.

## Materials and methods

### Reagents

SNH (MW: 330.41, purity≥98%) was purchased from Shanghai Yuanye Bio-technology Co. Ltd. (Shanghai, China). SNH was dissolved in 75 °C ddH_2_O as a 16 mmol/l stock solution and stored at 4 °C. Staurosporine (a PKC inhibitor) was purchased from Beyotime Biotech Inc. (Shanghai, China).

### Cell culture

NCI-H1299, A549, NCI-H460 and 293 T cells were obtained from the Stem Cell Bank, Chinese Academy of Sciences (Shanghai, China). SK-MES-1, SPC-A1 and HBE cells were kindly provided by Technology Transfer Center, NJUCM. 293 T, A549, SK-MES-1 and HBE cells were cultured in Dulbecco’s modified Eagle’s medium (DMEM) and F12 medium (Gibco, Australia), and NCI-H1299, NCI-H460 and SPC-A1 cells were cultured in RPMI 1640 medium (Gibco, Australia) with 10% foetal bovine serum (FBS; Gibco, Australia) supplemented with a 1% penicillin/streptomycin solution (Gibco, Australia). All of the cells were maintained at 37 °C in a humidified atmosphere with 5% CO_2_.

### Plasmid construction and cell transfection

A Linc00668 overexpression plasmid (p-Linc00668) and a negative control (NC) plasmid (p-NC) were designed by Realgene Biotech Co. (Nanjing, China). Three individual short hairpin RNA plasmids for Linc00668 (sh-Linc00668–1, sh-Linc00668–2, and sh-Linc00668–3) and a negative control (sh-NC) were purchased from Sangon Biotech (Shanghai, China) Co., Ltd. (Additional file 1: Table S2.). Hsa-miR-147a and NC mimics were purchased from Realgene Biotech Co. (Nanjing, China). Plasmids including the binding sites for miR-147a on Linc00668 and Slug mRNA were also designed by Realgene Biotech Co. (Nanjing, China). Cell transfection was performed with Lipofectamine 2000 transfection reagent (Invitrogen, US) according to the manufacturer’s protocol.

### Cell counting Kit-8 assay

The viability of NSCLC cells was assayed by Cell Counting Kit-8 (CCK-8; Dojindo, Beijing, China) assay. First, 5 × 10^3^ cells/well were added to a 96-well plate, with 100 μl of suspension in each well. Twenty-four hours after seeding, the medium was replaced with media containing different concentrations of SNH created through serial dilution. After culturing the cells for 24 H, 48 H, or 72 H, 10 μl of CCK-8 solution was added to each well, and the cells were incubated for 30 min. Curves of the cell death rates at the different SNH concentrations were calculated after measurement of the absorbance at each time point at a wavelength of 450 nm. The half maximal inhibitory concentration (IC_50_) values were also determined from these data.

### Transwell invasion assay and wound scratch assay

For the transwell cell invasion assay, 8.0 μm Transwell Permeable Supports 3422 (Corning, US) were used. The upper chambers were coated with Matrigel Basement Membrane Matrix 354,234 (Corning, US), and transfected or normal cells were seeded at a density of 5 × 10^3^ in the upper chambers with media containing different concentrations of SNH. A volume of 500 μl of medium containing 10% FBS was added to the lower chambers. After incubation for 24 H, the Matrigel and the cells on upper chambers were removed, and the cells on the bottom surface were fixed with 4% paraformaldehyde for 20 min. The cells were then stained with 0.3% crystal violet dye for 30 min. The invading cells were imaged using a digital light microscope (Leica, Germany).

For the wound scratch assay, cells were seeded at a density of 5 × 10^5^ cells/well in 6-well plates and exposed to the indicated treatments. After the cells reached 100% confluence, a sterilized 200 μl pipette tip was used to make a straight scratch in the middle of each well. Images were obtained by using digital light microscopy at each indicated time.

### Western blot analysis

Cells were lysed in radioimmunoprecipitation assay (RIPA) buffer (Solarbio, US) with 1% phenylmethanesulfonyl fluoride (PMSF). The lysates were suspended in sodium dodecyl sulfate-polyacrylamide gel electrophoresis (SDS-PAGE) sample loading buffer (Beyotime, Shanghai, China), separated on 10–15% SDS-PA gels (Beyotime, Shanghai, China) and transferred onto pure nitrocellulose blotting membranes (Pall, US). After the membranes were blocked with 5% non-fat milk, they were incubated at 4 °C overnight with primary antibodies against Slug (1:400, CST, US), E-cadherin (1:500, CST, US), N-cadherin (1:500, CST, US), Vimentin (1:1000, CST, US), and β-actin (1:2000, Santa Cruz, US). After incubation with horseradish peroxidase (HRP)-linked anti-rabbit (1:2000, CST, US) or anti-mouse (1:2000, CST, US) secondary antibodies for 2 h at room temperature, the bands were detected with a Gel Doc™ XR+ Gel Documentation System (Bio-Rad, US) with enhanced chemiluminescence (ECL) reagents (Bio-Rad, US).

### Quantitative real-time PCR (qRT-PCR)

Total RNA was isolated from cells and tissues using TRIzol reagent (Invitrogen, US) according to the manufacturer’s procedural guidelines. For mRNA and lncRNA quantification, the RNA was reverse transcribed into cDNA with a RevertAid First Strand cDNA Synthesis Kit (Thermo Fisher, US). EvaGreen 2X qRT-PCR MasterMix-Low ROX (abm, Canada) was used for quantitation with specific primers for the mRNA and lncRNA. GAPDH was used as an internal control. For miRNA quantification, reverse transcription was performed using an miRNA First Strand cDNA Synthesis (Stem-loop Method) Kit (Sangon Biotech, Shanghai, China). A MicroRNA qRT-PCR Kit (SYBR Green Method) (Sangon Biotech, Shanghai, China) was used for quantitation with specific primers for miRNA. U6 was used as an internal control. The primer sequences are listed in Additional file 2: Table S1. All quantitative real-time PCR experiments were performed with an Agilent Technologies Stratagene Mx3000P system (Agilent Technologies, US). The data were processed with the 2^-ΔΔCt^ method, and the fold changes were normalized to the expression of the internal controls.

### Immunofluorescence

Cells at a density of 1 × 10^4^ cells/well were seeded into 96-well plates. After exposure to the indicated treatments, the cells were fixed with 4% paraformaldehyde for 15 min at room temperature. Then, the cells were blocked with 5% goat serum and 0.3% Triton X-100 in phosphate-buffered saline (PBS) for 1 h. After the blocking solution was aspirated, the cells were incubated with a primary antibody against E-cadherin (1:200, CST, US), N-cadherin(1:200, CST, US), and Vimentin (1:100, CST, US) in antibody dilution buffer (ADB; 1X PBS/1% bovine serum albumin (BSA)/0.3% Triton X-100) overnight at 4 °C. The next day, the cells were incubated with a fluorochrome-conjugated anti-rabbit secondary antibody (1:1000, CST, US) in ADB for 2 h at room temperature in the dark. Subsequently, the cells were stained with DAPI (1 μg/ml, CST, US) for 5 min. Images were obtained under a fluorescence microscope.

When frozen sections were used for immunofluorescence, the sections were first blocked with goat serum; the rest of the procedure was the same as that for the goat serum-blocked cells.

### Dual-luciferase reporter assays

The target genes were inserted downstream of the firefly luciferase gene in the pmirGLO Dual-Luciferase miRNA Target Expression Vector (Promega, US). 293 T, NCI-H1299, and A549 cells were seeded in 24-well plates at a density of 1 × 10^4^ cells per well 24 H before transfection. The cells were co-transfected with 0.5 μg of pmirGLO vector and 20 pmol of mimics with 2 μl of Lipofectamine 2000 reagent (Invitrogen, US). After 36 h of transfection, the cells were lysed, and the firefly and Renilla luciferase activity was measured with a SpectraMax i3x microplate reader (Molecular Devices, US). The Renilla luciferase activity was used as an internal control.

### RNA immunoprecipitation (RIP) assay

The EZ-Magna RNA immunoprecipitation kit (Millipore, US) was used according to the manufacturer’s specifications. A549 and NCI-H1299 cells were lysed in complete RIP lysis buffer. The cell extract was then incubated with magnetic beads conjugated with Ago2 antibody (abcam, UK) or control IgG (Millipore, US) overnight at 4 °C. The beads were then washed and incubated with 0.1% SDS/0.5 mg/ml proteinase K for 30 min at 55 °C to remove proteins. Finally, the immunoprecipitated RNA was analysed by qRT-PCR analysis.

### Immunohistochemical (IHC) analysis

Tissue sections were dewaxed, dehydrated, and rehydrated. Then, citrate buffer was used for antigen retrieval, and hydrogen peroxide (3.0%) was used to block endogenous peroxidase activity. After blocking with 10% goat plasma, primary antibodies, including an antibody against Slug (1:400, ImmunoWay, US), were added to the sections and incubated at 4 °C overnight. SignalStain Antibody Diluent (CST, US) was used to detect the primary antibodies. Counterstaining was performed using haematoxylin.

### Lung metastasis in vivo

To assess the influence of SNH on the metastatic ability of NSCLC in vivo, we established a NSCLC orthotopic xenograft tumour model. First, we established the luciferase-expressing NCI-H1299 cell line with lentivirus (Ubi-MCS-firefly_Luciferase-IRES-Puromycin, GENE, Shanghai, China), and then NCI-H1299-luc cells (5 × 10^6^ in 0.2 ml medium of a 1:1 mixture of RPMI 1640 and Matrigel 354,248) were injected into left lung parenchyma of BALA/c nude mice. Two weeks later, the NSCLC model mice were identified and randomly divided into three groups that were treated with SNH (18.75, 37.5 or 75 mg/kg orally (p.o.) daily; *n* = 10) and a control group that was treated with olive oil (control, 100 μl, p.o. daily; *n* = 10) for 14 consecutive days. During this period, we monitored metastasis by luciferase imaging of live animals using an IVIS Spectrum bioluminescence imaging system (PerkinElmer, US) and the intraperitoneal injection of 200 μl D-Luciferin substrate (15 mg/ml in DPBS, PerkinElmer). Then, we collected half number of mice’ lungs in each group. The collected lung tissues were imaged with the IVIS system in the D-Luciferin substrate (150 μg/ml in DPBS) and analysed the expression of Linc00668 and slug with qRT-PCR. For the rest half number of mice, after the mice were subjected to vascular perfusion-fixation, the lungs were fixed with buffered formaldehyde solution. After paraffin-embedding the lungs, routine haematoxylin-eosin (H&E) staining and immunohistochemistry staining were performed to detect the basic lung morphology and target protein expression. Immunofluorescence of frozen sections was performed to evaluate the lung metastases. The protocols for the animal experiments were approved by the Ethics Review Committee of Nanjing University of Chinese Medicine.

### Statistical analysis

The data from the experiments are presented as the mean ± standard deviation (SD) of 3 independent experiments. Student’s t-test (two-tailed, with *p* < 0.05 considered significant) was used to assess differences between two groups. One-way analysis of variance, paired t-tests, χ2 tests or Spearman correlations were used to analyse multiple comparisons. We performed statistical analyses with GraphPad Prism 6 software.

## Result

### SNH beneficially reduces EMT progression in NSCLC cells

**Fig. 1:**
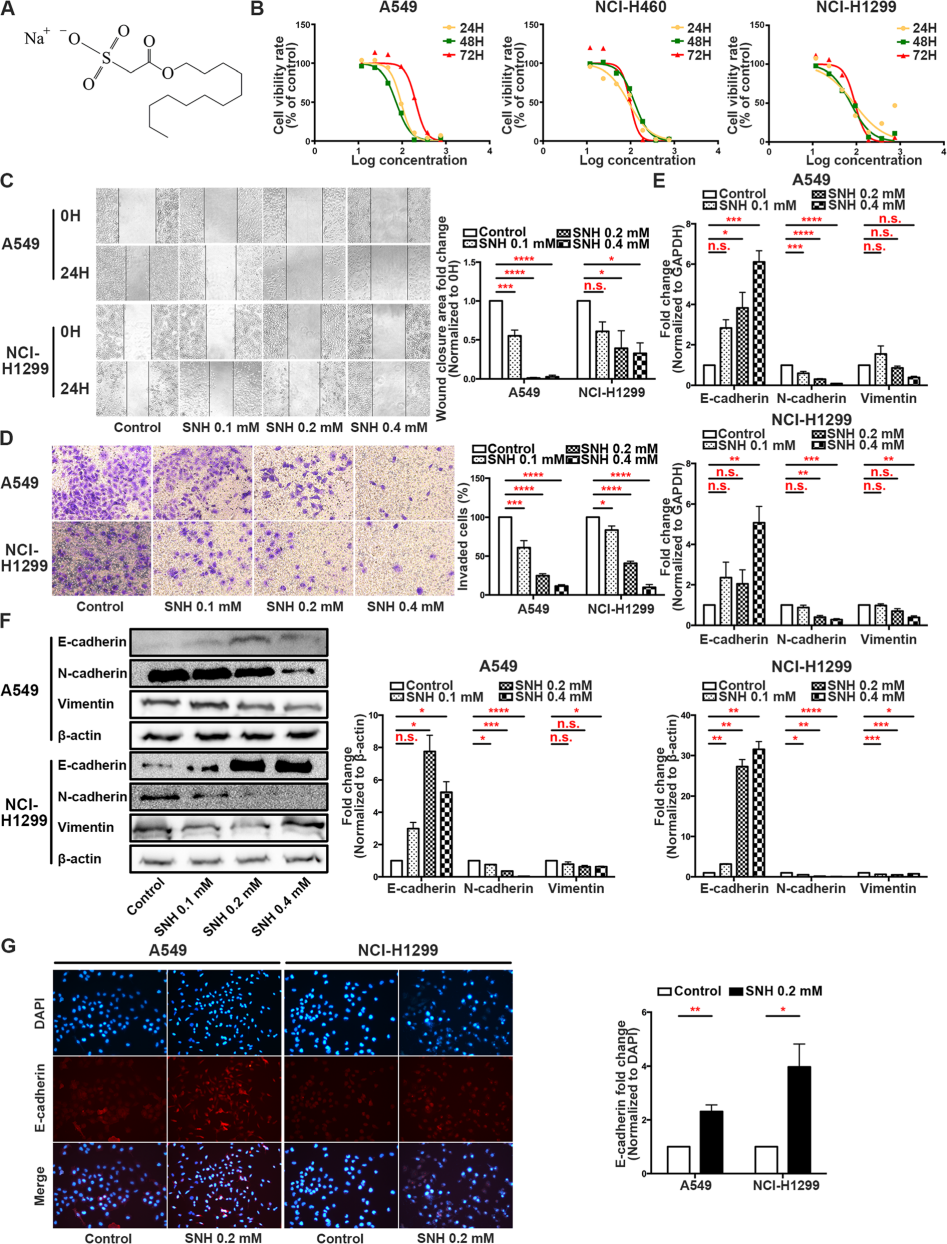
SNH beneficially reduces EMT progression in NSCLC cells. **a**. Chemical structure of SNH. **b**. CCK-8 proliferation assay of A549, NCI-H460 and NCI-H1299 cells treated with different concentrations of SNH for 24 H, 48 H or 72 H. **c**. Wound scratch assay of A549 and NCI-H1299 cells treated with SNH for 24 H. **d**. Transwell invasion assays showed that SNH decreased the invasion ability of A549 and NCI-H1299 cells. **e**. mRNA level of EMT markers in SNH-treated A549 and NCI-H1299 cells as determined by qRT-PCR. **f**. Expression of EMT markers in SNH-treated A549 and NCI-H1299 cells as determined by western blot analysis. **g**. Immunofluorescence staining of E-cadherin expression in SNH-treated A549 and NCI-H1299 cells. The bars and error bars indicate the mean ± SD. *p < 0.05, **p < 0.01, ***p < 0.005, ****p < 0.001

Metastasis is the leading clinical cause of cancer death. Chinese herbal medicine has multiple functions in inhibiting tumour cell metastasis. Before exploring the effect of SNH (structure shown in Fig. 1a) on NSCLC cells, we performed a CCK-8 assay to screen out the suitable concentration of the compound on NSCLC cell lines, and the results showed that 24 H of SNH treatment at 87.45~94.27 μmol/l had the best inhibitory effect on NSCLC cell proliferation (Fig. 1b). Under these conditions, we investigated whether SNH affected the migration and invasion ability of NSCLC cells in vitro. We treated A549 and NCI-H1299 cells with serial dilutions of SNH and then performed a wound scratch assay. The wound area was larger in cells that were treated with higher concentrations of SNH (Fig. 1c), which suggested that SNH inhibits NSCLC cell metastasis. To study the effects of SNH on invasion, we performed transwell invasion assays. After treating cells with SNH for 24 H, we determined that SNH decreased the invasion ability of the cells in a dose-dependent manner (Fig. 1d). For further investigation, we detected the expression of classic EMT markers via qRT-PCR. The results showed that A549 and NCI-H1299 cells treated with SNH had significantly higher E-cadherin expression and lower N-cadherin and Vimentin expression than control cells (Fig. 1e). Western blot analysis revealed that the expression of EMT-associated proteins was significantly modulated by SNH treatment (Fig. 1f). Immunofluorescence staining further revealed that SNH changed the E-cadherin, N-cadherin and Vimentin distribution in A549 and NCI-H1299 cells (Fig. 1g and Additional file 3: Figure S1A and B). These results demonstrated that SNH impeded metastasis by suppressing EMT progression in NSCLC cells.

### Linc00668 is a potential target of SNH

**Fig. 2:**
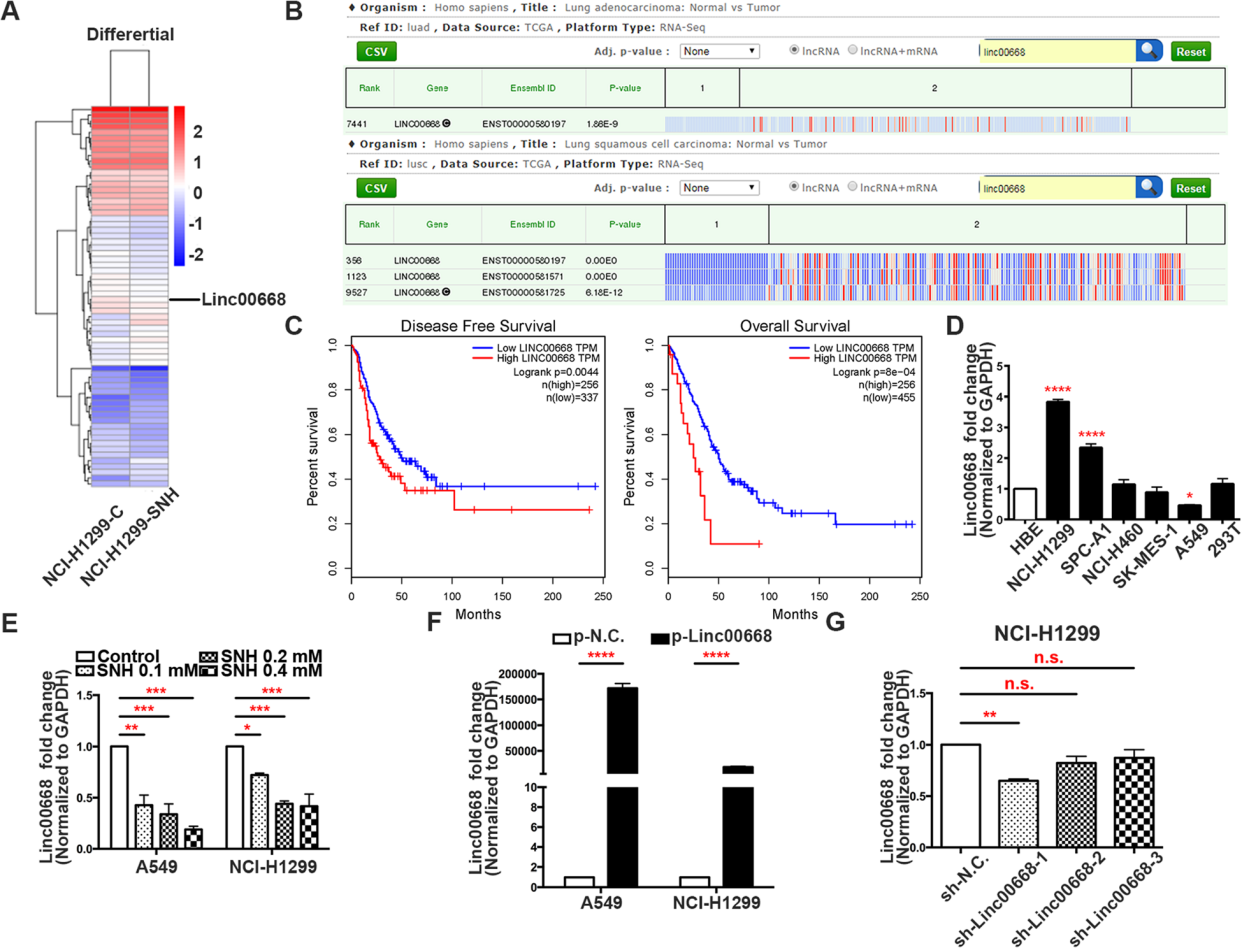
Linc00668 is a potential target of SNH. **a**. Heat map of the differential expression of screened lncRNA and mRNA sequences in NCI-H1299 cells treated or not treated with SNH for 24 H. **b**. Bioinformatics analysis of Linc00668 expression in human lung adenocarcinoma and lung squamous cell carcinoma tumour tissues compared with normal tissues. **c**. Association of Linc00668 expression with NSCLC patient disease-free survival and overall survival. **d**. Linc00668 expression in 5 NSCLC cell lines, 1 human bronchial epithelial cell line (HBE) and 1 human renal epithelial cell line (293 T) as determined by qRT-PCR. **e**. Linc00668 expression in SNH-treated A549 and NCI-H1299 cells as determined by qRT-PCR. **f**. Validation of Linc00668 overexpression by p-Linc00668 in A549 and NCI-H1299 cells as determined by qRT-PCR. **g**. Validation of sh-RNA knockdown efficiency in NCI-H1299 cells as determined by qRT-PCR. The bars and error bars indicate the mean ± SD. *p < 0.05, **p < 0.01, ***p < 0.005, ****p < 0.001

In an attempt to identify the lncRNA required for the activity of SNH in NSCLC, we screened lncRNA and mRNA sequences. In a comparison of NCI-H1299 cells treated or not treated with SNH, a transcriptome analysis with GO terms indicated that SNH may negatively regulated cell migration or motility (Additional file 4: Figure S2A), and a pathway analysis using the KEGG pathway database suggested that SNH influences genes in EMT-associated pathways, such as the wnt signalling pathway and TGF-β signalling pathway (Additional file 4: Figure S2B). Heat maps showed a clear distinction between the groups, with Linc00668 expression significantly decreased in NSCLC cells treated with SNH (Fig. 2a). Using the bioinformatics tool “lncRNAtor”, we found that Linc00668 was expressed at significantly higher levels in NSCLC tumour tissues than in normal tissues (Fig. 2b). An analysis of data from the Cancer Genome Atlas (TCGA) demonstrated that higher Linc00668 levels in NSCLC patients were always associated with worse overall survival and disease-free survival outcomes (Fig. 2c). Thus, we suspected that Linc00668 was a target influenced by SNH. To investigate the functional role of Linc00668 in NSCLC cells, we first performed qRT-PCR analysis to examine the expression of Linc00668 in a diverse range of human NSCLC cell lines. Linc00668 was expressed at significantly higher levels in NCI-H1299 and SPC-A1 cells (Fig. 2d), especially in NCI-H1299 cells, than in normal human HBE cells, while it showed lower expression in A549 cells (Fig. 2d). Regarding cells that were treated with SNH, the qRT-PCR results showed that the level of Linc00668 in NCI-H1299 and A549 cells decreased with increasing SNH concentrations (Fig. 2e). To further investigate the role of Linc00668 in NSCLC cells, we constructed a Linc00668 overexpression vector using pIRES2-EGFP, and after transfection of p-Linc00668 into NCI-H1299 and A549 cells, the expression level of Linc00668 was significantly elevated (Fig. 2f). In contrast, we used pSGU6/GFP/Neo to construct 3 kinds of Linc00668-knockdown plasmids, named sh-Linc00668–1, sh-Linc00668–2 and sh-Linc00668–3. Upon evaluation of the cell transfection efficiency, the knockdown efficiency of si-Linc00668–1 was found to be the best (Fig. 2g), and we used si-Linc00668–1 to conduct the following experiments.

### Linc00668 is required for the EMT in NSCLC cells

**Fig. 3:**
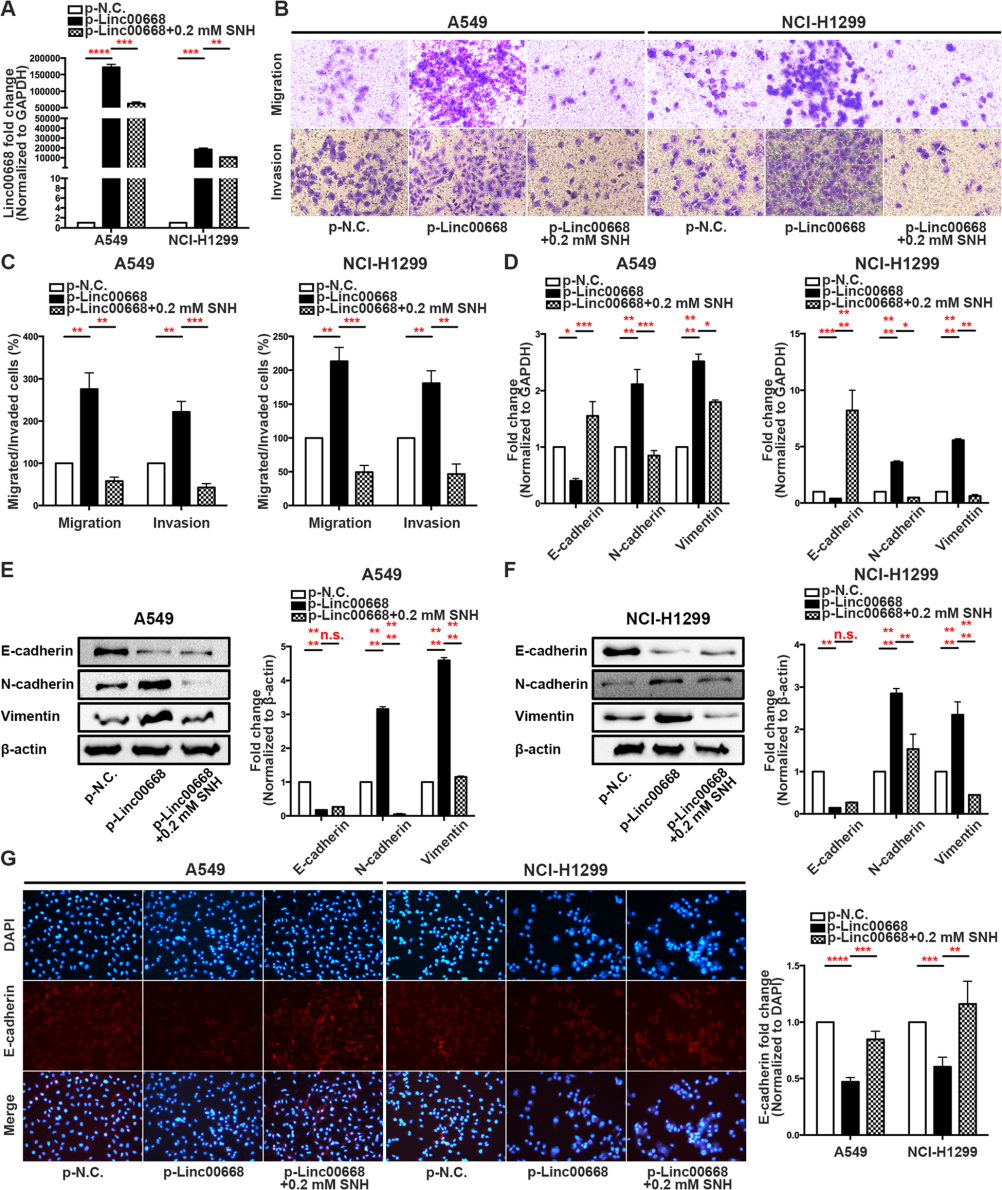
Linc00668 is required for the EMT in NSCLC cells. **a**. Linc00668 expression in SNH-treated A549 and NCI-H1299 cells after p-Linc00668 transfection as determined by qRT-PCR. **b-c**. Transwell invasion/migration assays showed that the metastatic ability of A549 and NCI-H1299 cells was increased after p-Linc00668 transfection but was diminished after the addition of SNH. **d**. mRNA levels of EMT markers in SNH-treated A549 and NCI-H1299 cells after p-Linc00668 transfection as determined by qRT-PCR. **e-f**. Expression of EMT markers in SNH-treated A549 (**e**) and NCI-H1299 (**f**) cells after p-Linc00668 transfection as determined by western blot analysis. **g**. Immunofluorescence staining of E-cadherin expression in SNH-treated A549 and NCI-H1299 cells after p-Linc00668 transfection. The bars and error bars indicate the mean ± SD. *p < 0.05, **p < 0.01, ***p < 0.005, ****p < 0.001

To investigate the relationship between Linc00668, SNH and the EMT, we first overexpressed Linc00668 in cells and treated them with SNH. QRT-PCR showed that Linc00668 was significantly decreased after treatment with SNH (Fig. 3a). Functionally, transwell assays demonstrated that overexpression of Linc00668 significantly potentiated the migration and invasion ability of A549 and NCI-H1299 cells; however, after treatment with SNH, the cell migration and invasion ability was diminished (Fig. 3b and c). Moreover, in qRT-PCR and western blot assays on A549 and NCI-H1299 cells overexpressing Linc00668, we observed a decrease in the expression of the epithelial marker E-cadherin accompanied by an increase in the mesenchymal markers Vimentin and N-cadherin; however, after the cells were treated with SNH, the results were reversed (Fig. 3d, e and f). Immunofluorescence staining revealed that Linc00668 overexpression changed the E-cadherin, Vimentin and N-cadherin distribution in the absence or presence of SNH in A549 and NCI-H1299 cells. The fluorescence intensity suggested that E-cadherin expression decreased when Linc00668 was overexpressed but increased when SNH was added; however, opposite results were observed for Vimentin and N-cadherin (Fig. 3g and Additional file 5: Figure S3A and B). Consistent with this result, Linc00668 silencing also diminished the cell migration and invasion of NCI-H1299 cells in transwell assays (Additional file 5: Figure S3C). The qRT-PCR and western blotting results revealed that Linc00668 silencing significantly upregulated E-cadherin and downregulated N-cadherin and Vimentin at the mRNA and protein levels in NCI-H1299 cells (Additional file 5: Figure S3D and E). Further immunofluorescence experiments showed that silencing Linc00668 increased the fluorescence intensity of E-cadherin and decreased the fluorescence intensity of N-cadherin and Vimentin in NCI-H1299 cells (Additional file 5: Figure S3F, G and H). In conclusion, Linc00668, which could be suppressed by SNH, was required as a key gene for the progression of the EMT in NSCLC cells.

### Linc00668 functions as a ceRNA and sponges miR-147a in NSCLC cells

**Fig. 4:**
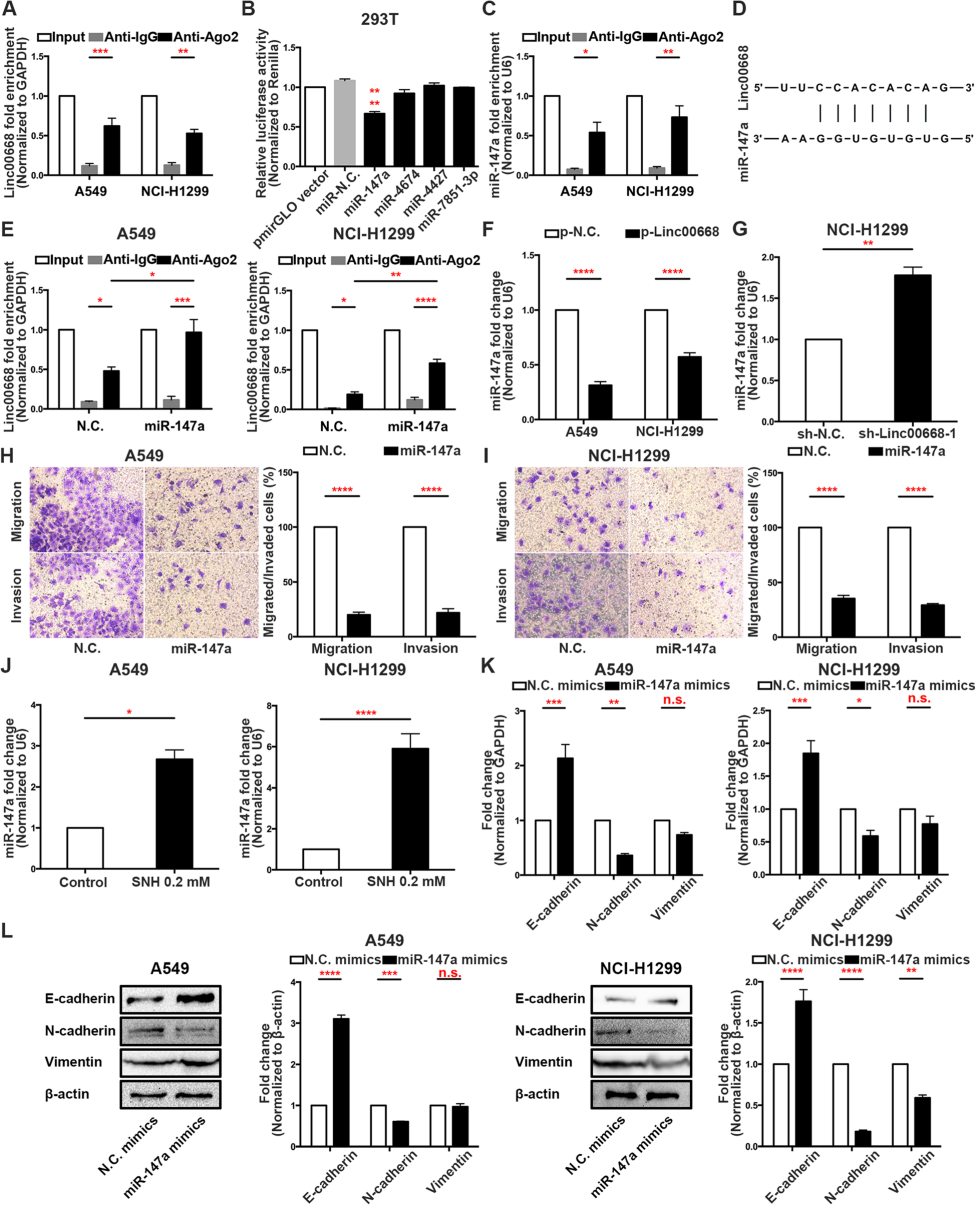
Linc00668 functions as a ceRNA and sponges miR-147a in NSCLC cells. **a**. RIP assays of Linc00668 binding to Ago2 in A549 and NCI-H1299 cell extracts. **b**. Dual-luciferase reporter assays were used to determine the interaction between miRNAs and Linc00668. **c**. RIP assays of miR-147a binding to Ago2 in A549 and NCI-H1299 cell extracts. **d**. Predicted binding sites for miR-147a in the linc00668 sequence. **e**. RIP assays of Linc00668 binding to Ago2 in miR-147a mimic-transfected A549 and NCI-H1299 cell extracts. **f**. MiR-147a expression in p-Linc00668-transfected A549 and NCI-H1299 cells as determined by qRT-PCR. **g**. MiR-147a expression in sh-Linc00668–1-transfected NCI-H1299 cells as determined by qRT-PCR. **h-i**. Transwell invasion/migration assays showed that the metastatic ability of A549 (**h**) and NCI-H1299 (**i**) cells was diminished after miR-147a mimic transfection. **j**. MiR-147a expression in SNH-treated A549 and NCI-H1299 cells as determined by qRT-PCR. **k**. mRNA levels of EMT markers in miR-147a mimic-transfected A549 and NCI-H1299 cells as determined by qRT-PCR. **L**. Expression of EMT markers in miR-147a mimic-transfected A549 and NCI-H1299 cells as determined by western blot analysis. The bars and error bars indicate the mean ± SD. *p < 0.05, **p < 0.01, ***p < 0.005, ****p < 0.001

Some studies have declared that lncRNAs exert effects not only by epigenetically regulating targets in the nucleus but also by functioning as ceRNAs for particular miRNAs [18]. Interestingly, the RIP assay showed that Linc00668 could bind the Ago2 protein, suggesting that it could be acting as a ceRNA (Fig. 4a). With a help of the miRNA target prediction tool DIANA, we predicted that miR-147a, miR-4674, miR-4427 and miR-7851-3p could be targets of Linc00668. Dual-luciferase reporter assays showed that the ability of Linc00668 to bind miR-147a was significantly stronger than its ability to bind the other miRNAs (Fig. 4b). A RIP assay verified that miR-147a was preferentially enriched among Ago2-containing microRNA ribonucleoproteins (miRNPs) compared to IgG (Fig. 4c). Moreover, the bioinformatics tool also found the binding sites of Linc00668 and miR-147a (Fig. 4d), confirming that Linc00668 could act as a ceRNA for miR147a in NSCLC cells. Consistently, after transfection of cells with miR-147a mimics, the RIP assay revealed increased enrichment of Linc00668 on anti-Ago2-beads (Fig. 4e). Furthermore, miRNA qRT-PCR analysis showed that miR-147a expression was diminished upon overexpression of Linc00668 (Fig. 4f) and was potentiated upon silencing of Linc00668 (Fig. 4g). To investigate whether miR-147a played a role in the SNH anti-NSCLC mechanism, we analysed the miR-147a fold changes after SNH treatment in A549 and NCI-H1299 cells. As expected, miR-147a expression increased with increasing SNH (Fig. 4j). To further investigate the relationship between miR-147a and the EMT in NSCLC, we transfected miR-147a mimics into A549 and NCI-H1299 cells. The results revealed that the migration and invasion ability of A549 and NCI-H1299 cells was markedly weakened by miR-147a overexpression (Fig. 4h and i). Next, qRT-PCR, western blotting and immunofluorescence demonstrated that miR-147a overexpression prevented EMT progression in NSCLC cells on the levels of nucleic acid expression, protein expression and protein distribution (Fig. 4k, l and Additional file 6: Figure S4A, B and C). Therefore, we concluded that miR-147a could inversely regulate the EMT in NSCLC cells.

### MiR-147a directly targets slug to mediate EMT progression in NSCLC cells

**Fig. 5:**
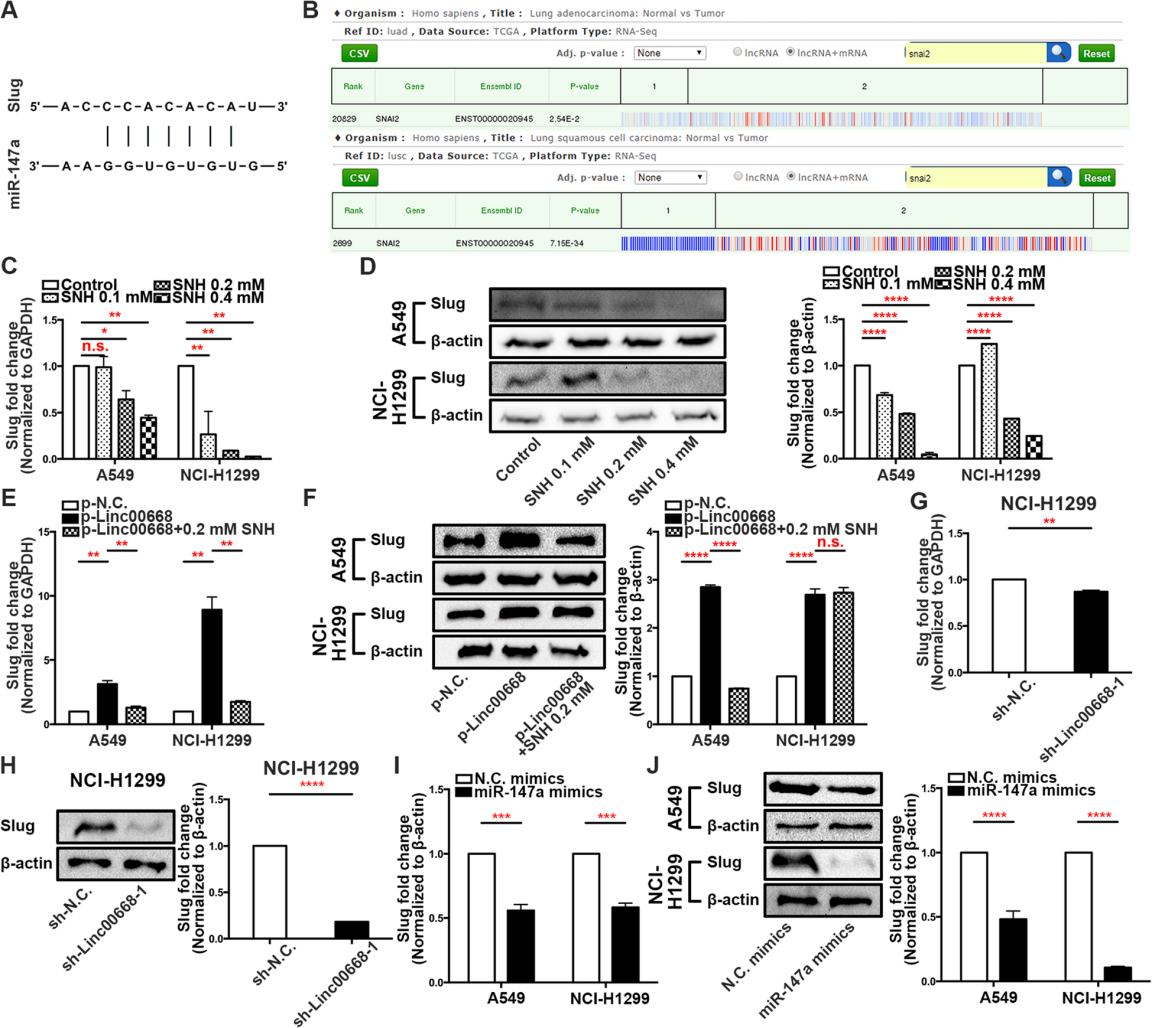
MiR-147a directly targets Slug to mediate EMT progression in NSCLC cells. **a**. Predicted binding sites for miR-147a in the slug mRNA sequence. **b**. Bioinformatics analysis of Slug expression in human lung adenocarcinoma and lung squamous cell carcinoma tumour tissues compared with normal tissues. **c-d**. Slug expression in SNH-treated A549 and NCI-H1299 cells as determined by qRT-PCR (**c**) and western blot analysis (**d**). **e-f**. Slug expression in SNH-treated A549 and NCI-H1299 cells after p-Linc00668 transfection as determined by qRT-PCR (**e**) and western blot analysis (**f**). **g-h**. Slug expression in sh-Linc00668–1-transfected NCI-H1299 cells as determined by qRT-PCR (**g**) and western blot analysis (**h**). **i-j**. Slug expression in miR-147a mimic-transfected A549 and NCI-H1299 cells as determined by qRT-PCR (**i**) and western blot analysis (**j**). The bars and error bars indicate the mean ± SD. *p < 0.05, **p < 0.01, ***p < 0.005, ****p < 0.001

To further elucidate the molecular mechanism underlying how miR-147a exerted its effect on the EMT in NSCLC cells, we used bioinformatics tools to predict candidate downstream targets. Binding sequences for miR-147a were identified in the 3′ UTR of Slug mRNA (Fig. 5a). Slug is considered a classic transcription factor in EMT progression [22]. In the TCGA dataset, the expression of Slug was highly expressed in both lung adenocarcinoma and lung squamous cell carcinoma (Fig. 5b). Therefore, Slug is an important index to assess the migration and invasion of NSCLC. QRT-PCR and western blot analysis confirmed that SNH dose-dependently inhibited Slug expression in A549 and NCI-H1299 cells (Fig. 5c and d). In addition, while Slug was highly expressed upon overexpression of Linc00668, its expression decreased with the addition of SNH (Fig. 5e and f). Furthermore, Slug expression was diminished upon the silencing of Linc00668 at both the mRNA and protein levels (Fig. 5g and h). Additionally, qRT-PCR and western blotting revealed that miR-147a could negatively modulate the expression of Slug in A549 and NCI-H1299 cells (Fig. 5i and j). In summary, we concluded that Slug was a direct target of miR-147a and was positively modulated by Linc00668 in NSCLC cells.

### Linc00668 regulated slug expression indirectly via sponging miR-147a

**Fig. 6:**
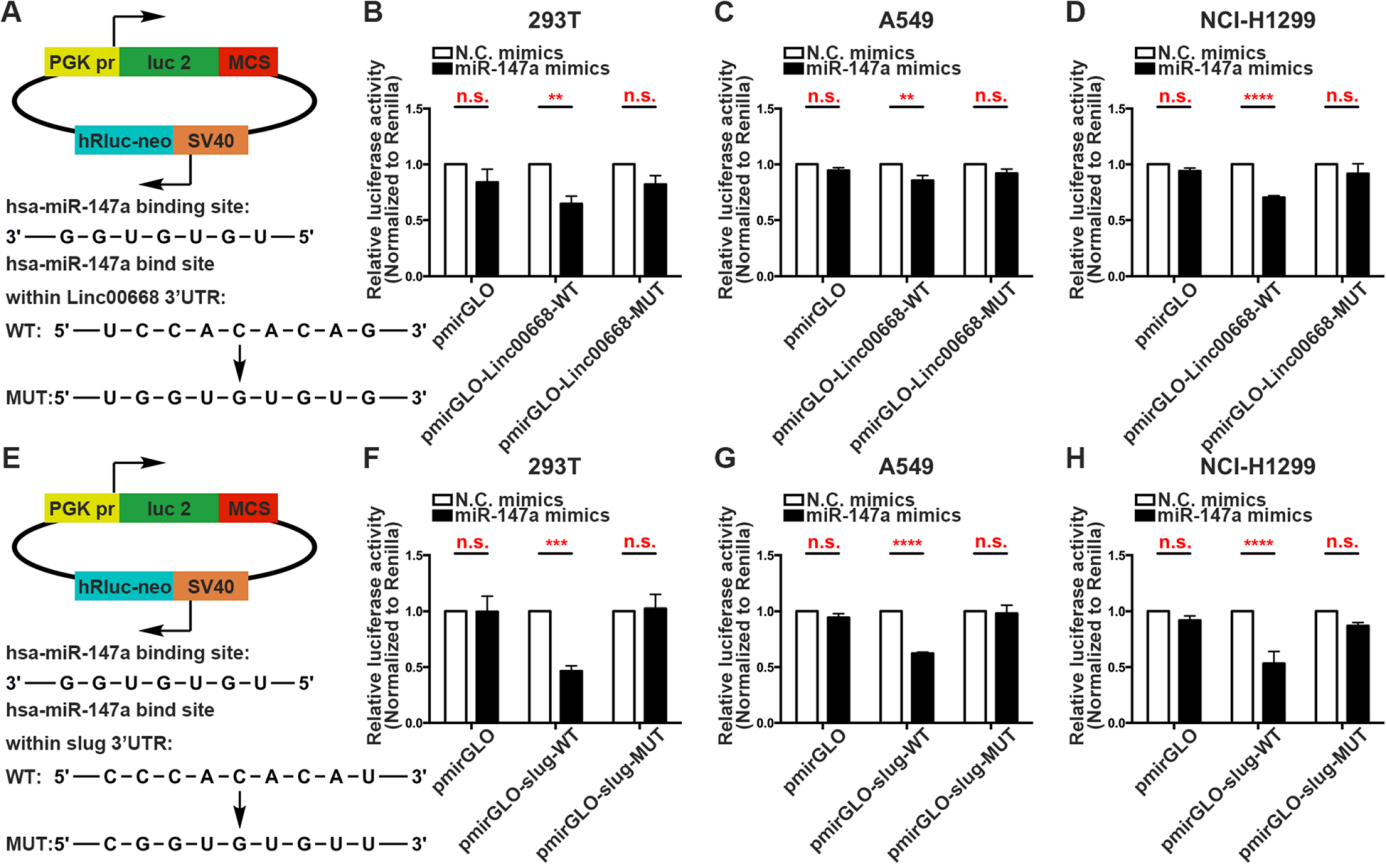
Linc00668 regulated Slug expression indirectly via sponging miR-147a. **a**. Predicted binding sites for miR-147 on Linc00668 and a diagram depicting the construction of the wild type (WT) and mutant type (MUT) pmirGLO-Linc00668 plasmids. **b-d**. 293 T (**b**), A549 (**c**) and NCI-H1299 (**d**) cells were co-transfected with miR-147a mimics or N.C. mimics and pmirGLO or pmirGLO-Linc00668-WT or pmirGLO-Linc00668-MUT. Luciferase activity was detected 24 H after transfection using a dual-luciferase assay. **e**. Predicted binding sites for miR-147 on slug and a diagram depicting the construction of the WT and MUT pmirGLO-slug plasmids. **f-h**. 293 T (**f**), A549 (**g**) and NCI-H1299 (**h**) cells were co-transfected with miR-147a mimic or N.C. mimics and pmirGLO or pmirGLO-slug-WT or pmirGLO-slug-MUT. Luciferase activity was detected 24 H after transfection using a dual-luciferase assay. The bars and error bars indicate the mean ± SD. *p < 0.05, **p < 0.01, ***p < 0.005, ****p < 0.001

CeRNAs have emerged as important factors in the lncRNA and miRNA regulatory network [20]. To investigate the miR-147a binding sites on Linc00668 and Slug, we performed dual-luciferase reporter assays for further transcriptional analysis. First, we sub-cloned the wild-type (WT) or mutated binding region (predicted to have miR-147a binding sites; Additional file 7: Table S3) of Linc00668 downstream of the firefly luciferase gene in the pmirGLO vector (Fig. 6a). We then evaluated luciferase activity after co-transfection of cells with miRNA mimics and luciferase plasmids. In 293 T, A549 and NCI-H1299 cells, we observed that overexpression of miR-147a significantly reduced the luciferase activity of pmirGLO-Linc00668-WT while mutation in the miR-147a binding sites restored luciferase activity (Fig. 6b-d). Then, we constructed pmirGLO dual-luciferase reporter vectors with WT or mutated Slug 3′ UTRs (predicted to have miR-147a binding sites; Additional file 7: Table S3; Fig. 6e), and reporter assays were performed. Compared with the NC groups, the groups of 293 T, A549 and NCI-H1299 cells co-transfected with pmirGLO-Slug-WT vector and miR-147a mimics showed remarkably diminished luciferase activity (Fig. 6f-h). Together, these results demonstrated that Linc00668 regulated Slug expression indirectly via sponging miR-147a.

### Verification that SNH suppresses the EMT in vivo

**Fig. 7:**
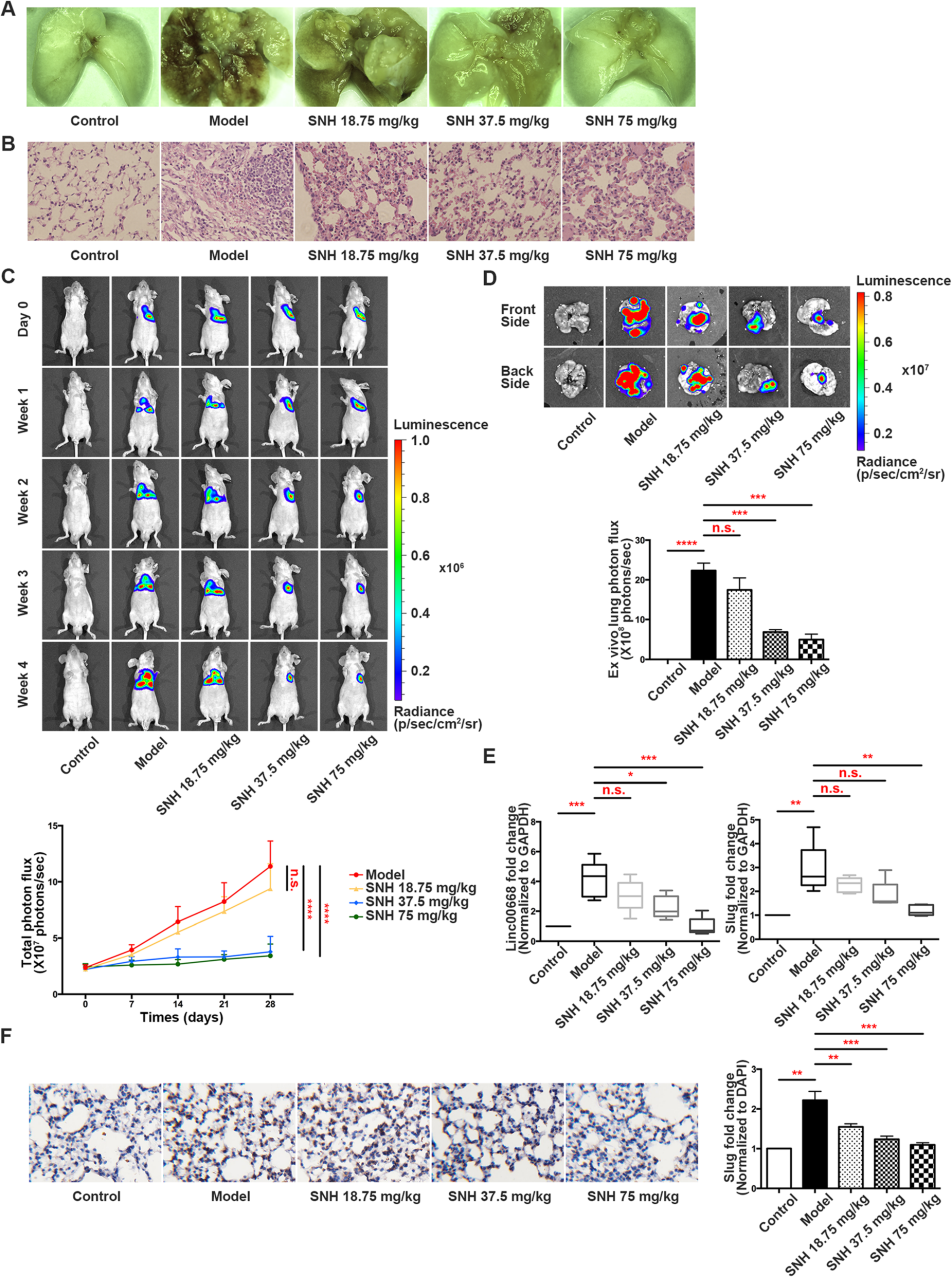
Verification that SNH suppresses the EMT in vivo. **a**. Representative images of the lungs of nude mice that were injected with NCI-H1299-luc cells and treated with SNH. **b**. Representative images showing haematoxylin and eosin staining of lung samples from the different groups. **c**. Bioluminescent imaging and quantification of photon flux of different dose of SNH influenced groups with left lung parenchyma injection of luciferase-marked NCI-H1299 cells. **d**. Bioluminescent imaging and quantification of the photon flux of lungs from different groups. **e**. Linc00668 and slug expression in the tissue of lungs of the different groups as determined by qRT-PCR. **f**. IHC staining showing slug expression in the different groups. The bars and error bars indicate the mean ± SD. *p < 0.05, **p < 0.01, ***p < 0.005, ****p < 0.001

To analyse the relationship between SNH and the EMT in vivo, we first injected NCI-H1299-luc cells into the left lung parenchyma of BALA/c nude mice and then treated the mice with different concentrations of SNH. Lung tissues were extracted as samples for the following study. Stereological observations and H&E staining of the pathological sections showed that the lung tissue was cancerous after the model was established, but the symptoms were relieved after treatment with SNH (Fig. 7a and b). In bioluminescent images, dissemination and metastasis in the luciferase tracking NSCLC cells were suppressed by SNH especially in the concentration of 37.5 and 75 mg/kg (Fig. 7c and d). The qRT-PCR assay revealed that the expression of Linc00668 and Slug in the tissue of lungs was decreased after SNH treatment in the test groups (Fig. 7e). Immunofluorescence of frozen sections showed that the fluorescence intensity of the epithelial marker E-cadherin was positively associated with the concentration of SNH (Additional file 8: Figure S5A), which illustrated that the migration and invasion ability of lung cancer was decreased by SNH. IHC staining revealed that Slug was diminished by SNH treatment (Fig. 7f). Overall, EMT progression was activated with the establishment of the metastatic lung cancer model but was attenuated after SNH gavage. Therefore, we deduced that SNH could suppress NSCLC migration and invasion in vivo.

## Discussion

An increasing number of research studies have focused on the anticancer mechanisms of NPs in recent years [23]. In an analysis of all approved anticancer drugs worldwide, 74.9% were found to be either NPs per se or naturally inspired agents [24]. Compared to chemically synthesized compounds, NPs are more frequently clinically used [7]. SNH, a naturally inspired compound of *Houttuynia cordata*, has traditionally been used to treat lung disease in Chinese medicine. For instance, Chen YF et al. demonstrated that *Houttuynia cordata* Thunb. induced apoptosis in A549 cells [25].

Metastasis is a basic characteristic of lung cancer. Recent studies have indicated that EMT progression is part of a classic theory of metastasis [26]. Studies on the NPs that regulate EMT progress in lung cancer are currently ongoing. Avtanski DB et al. reported the specific pathways of HNK (Honokiol, a natural phenolic compound)-mediated EMT inhibition for breast carcinoma [27]. Because we successfully confirmed that SNH could repress EMT progression, a further study of the mechanism was needed to support this phenomenon.

The sheer number and diversity of lncRNAs juxtaposed with their complex molecular functions in editing, modification, retrotransposition and inheritance [28] suggests that scientists are far from understanding the interconnected relationships among lncRNAs and biological processes. Considering that an increasing number of lncRNAs have been identified as regulators of migration and invasion in NSCLC cells, we strived to determine whether there was an intimate relationship between lncRNAs and changes in metastasis after SNH treatment in NSCLC cells. After the NSCLC cells were treated with SNH, a screening of the lncRNA and mRNA sequences was performed, and GO and KEGG analyses confirmed that the EMT-associated pathway was influenced and the expression of Linc00668 was a potential target. Genomics and survival analyses with the TCGA database showed that Linc00668 expression was significantly elevated in NSCLC samples and was associated with poor prognosis. Thus, we proposed that Linc00668 might be a tumour promoter in NSCLC.

Recent studies on Linc00668 focused on proliferation and poor prognosis. Zhang CZ observed that LINC00668 exerts its oncogenic effects in oral squamous cell carcinoma (OSCC) partially via sponging miR-297 and activating VEGFA, which may be a negative prognostic factor [29]. Zhang E et al. declared that LINC00668 was a directly regulated target of E2F1, which may be a downstream effector that binds to PRC2 in gastric cancer (GC) [30]. To further investigate the biological function of Linc00668, we constructed knockdown and overexpression models. The results showed that Linc00668 could encourage the metastasis of NSCLC cells. However, when cells overexpressing Linc00668 were treated with SNH, migration and invasion were diminished, as was the expression of Linc00668, which confirmed that SNH suppresses metastasis by inhibiting Linc00668.

The ceRNA theory of lncRNA has been extensively accepted by increasing numbers of studies [31–34]. In bioinformatics prediction of Linc00668, 4 possible binding miRNAs were identified, and miR-147a was considered the most likely candidate. Confirmation of the ability of Linc00668/miR-147a to bind to the Ago2 protein by RIP assay further illuminated the role of miR-147a. Convincingly, miR-147a-associated metastasis experiments showed that overexpression of miR-147a promoted cell migration and invasion. Recent studies on miR-147a have focused only on proliferation [35, 36]. Bertero T et al. reported that miR-147a appeared to be a potent inhibitor of cell proliferation and migration [37], but there is a lack of subsequent data to explain the mechanism of miR-147a in metastasis. Encouragingly, in the current study, the 3 ‘UTR region of Slug was predicted to partly bind with miR-147a. Slug is a common zinc finger transcriptional repressor that downregulates the expression of E-cadherin and triggers metastasis [38]. The qRT-PCR and western blot analyses confirmed that Slug was downregulated by SNH. Overall, we verified that SNH negatively regulates metastasis of NSCLC in vitro and in vivo by the Linc00668/miR-147a/Slug axis.

## Conclusions

**Fig. 8:**
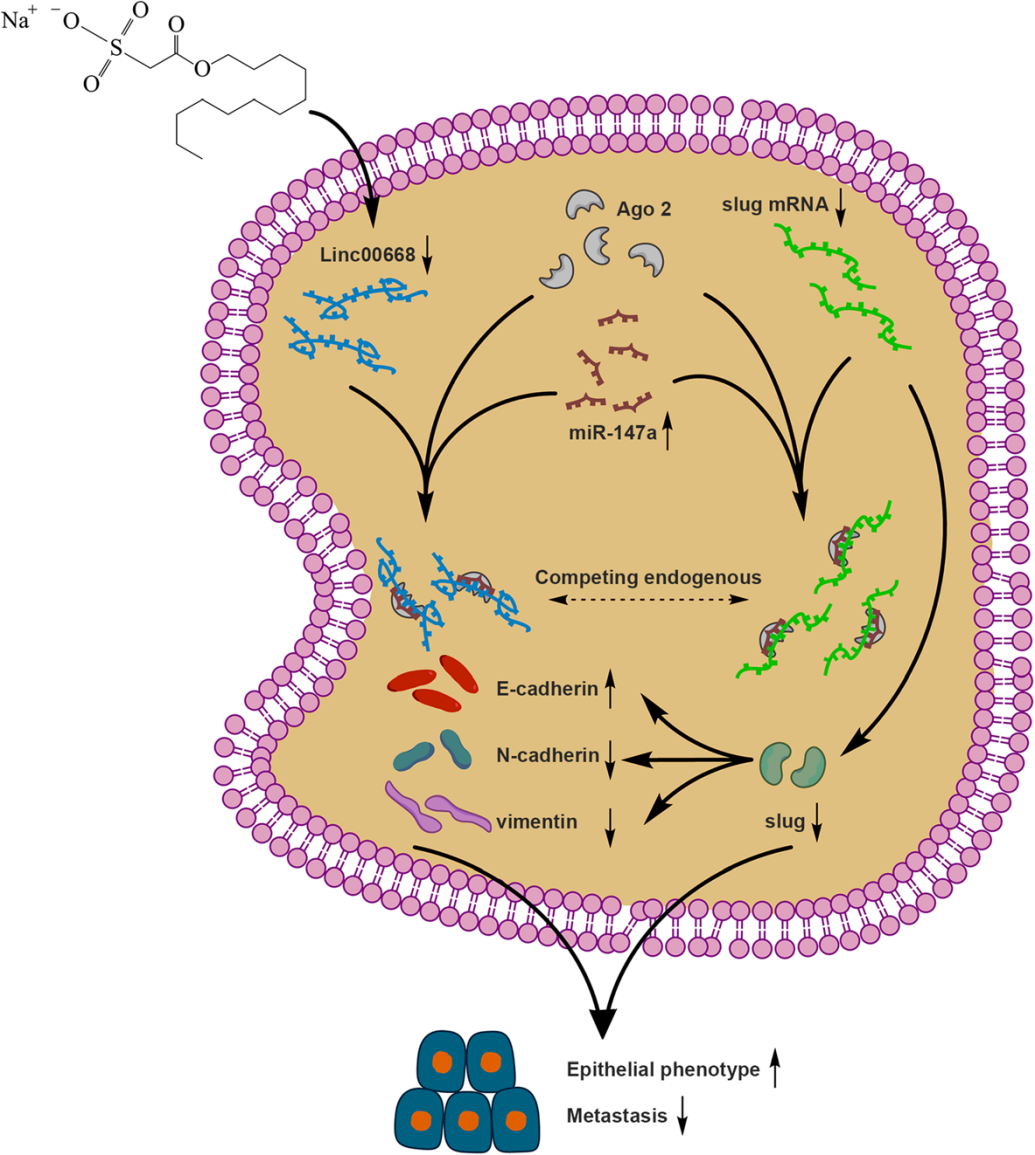
Schematic diagram of mechanism on this research. SNH negatively regulates metastasis of NSCLC by Linc00668/miR-147a/Slug axis

Our present study demonstrated for the first time that SNH can suppress metastasis of NSCLC cell. The mechanism of metastasis regulation may involve the Linc00668/miR-147a/Slug axis (Fig. 8). Our findings provide support for the clinical use of SNH in NSCLC patients and provide a potential molecular therapeutic strategy.

## Additional files

Additional file 1: **Table S2.** The sh-RNA sequences. (PDF 40 kb)

Additional file 2: **Table S1.** Primers for qRT-PCR. (PDF 36 kb)

Additional file 3: **Figure S1 A-B.** Immunofluorescence staining of N-cadherin (A) and Vimentin (B) expression in SNH-treated A549 and NCI-H1299 cells. The bars and error bars indicate the mean ± SD. *p < 0.05, **p < 0.01, ***p < 0.005, ****p < 0.001. (TIF 1632 kb)

Additional file 4: **Figure S2 A.** Transcriptome analysis, which was based on GO terms, of the screened lncRNA and mRNA sequences in NCI-H1299 cells treated or not treated with SNH for 24 H. **B**. Pathway analysis, which was based on the KEGG pathway database, of the screened lncRNA and mRNA sequences in NCI-H1299 cells treated or not treated with SNH for 24 H. (TIF 373 kb)

Additional file 5: **Figure S3,. A-B**. Immunofluorescence staining of N-cadherin (**A**) and Vimentin (**B**) expression in SNH-treated A549 and NCI-H1299 cells after p-Linc00668 transfection. **C**. Transwell invasion/migration assays showed that the metastatic ability of NCI-H1299 cells was increased after sh-Linc00668–1 transfection. **D**. mRNA levels of EMT markers in sh-Linc00668–1-transfected NCI-H1299 cells as determined by qRT-PCR. **E**. Expression of EMT markers in sh-Linc00668–1-transfected NCI-H1299 cells as determined by western blot analysis. **F-H**. Immunofluorescence staining of E-cadherin (**F**), N-cadherin (**G**) and Vimentin (**H**) expression in sh-Linc00668–1-transfected NCI-H1299 cells. The bars and error bars indicate the mean ± SD. *p < 0.05, **p < 0.01, ***p < 0.005, ****p < 0.001. (TIF 4296 kb)

Additional file 6: **Figure S4. A-C**. Immunofluorescence staining of E-cadherin (**A**), N-cadherin (**B**) and Vimentin (**C**) expression in miR-147a mimic-transfected A549 and NCI-H1299 cells. The bars and error bars indicate the mean ± SD. *p < 0.05, **p < 0.01, ***p < 0.005, ****p < 0.001. (TIF 2341 kb)

Additional file 7: **Table S3.** Dual-luciferase report assay vectors. (PDF 34 kb)

Additional file 8: **Figure S5. A**. Immunofluorescence staining of frozen sections showing E-cadherin expression in the different groups. The bars and error bars indicate the mean ± SD. *p < 0.05, **p < 0.01, ***p < 0.005, ****p < 0.001. (TIF 4677 kb)

## Abbreviations

3′-UTR: 3′-untranslated region;
CeRNA: Competing endogenous RNA
EMT: Epithelial-mesenchymal transition
IHC assay: Immunohistochemical assay
LncRNAs: Long non-coding RNAs
MiRNAs: MicroRNAs
NPs: Natural products
NSCLC: Non-small cell lung cancer
QRT-PCR: Quantitative real-time PCR
RIP assay: RNA immunoprecipitation assay
SD: Standard deviation
SNH: Sodium new houttuyfonate
TCGA: The Cancer Genome Atlas
