# Supplementary material for: Sodium new houttuyfonate suppresses metastasis in NSCLC cells through the Linc00668/miR-147a/slug axis
Source: J Exp Clin Cancer Res. 2019 Apr 11;38:155. doi: 10.1186/s13046-019-1152-9 (PMC6458838; doi:10.1186/s13046-019-1152-9)
Supplement: Supplementary file 1 — Table S2. The sh-RNA sequences. (PDF 40 kb) [file 13046_2019_1152_MOESM1_ESM.pdf]

Table S1. The sh-RNA sequences

Gene	Target position	Sh-RNA sequences
**Sh-NC-sense**		5'-CACCGTTCTCCGAACGTGTCACGTCAAGAGATTACGTGACACGTTCGGAGAATTTTTTG-3
**Sh-NC-antisense**		5'-AGCTCAAAAAATTCTCCGAACGTGTCACGTAATCTCTTGACGTGACACGTTCGGAGAAC-3’
**Sh-Linc00668-1-sense**	180-202	5'-CACCGCTGAAGCAGCATCACTGTCTTTCAAGAGAAGACAGTGATGCTGCTTCAGCTTTTTTG-3’
**Sh-Linc00668-1-antisense**		5'-AGCTCAAAAAAGCTGAAGCAGCATCACTGTCTTCTCTTGAAAGACAGTGATGCTGCTTCAGC-3’
**Sh-Linc00668-2-sense**	508-530	5'-CACCGCTTCAAGTTTCATTCTCTCTTTCAAGAGAAGAGAGAATGAAACTTGAAGCTTTTTTG-3’
**Sh-Linc00668-2-antisense**		5'-AGCTCAAAAAAGCTTCAAGTTTCATTCTCTCTTCTCTTGAAAGAGAGAATGAAACTTGAAGC-3’
**Sh-Linc00668-3-sense**	742-764	5'-CACCGTAATCTGGCACGTATAGTTCTTCAAGAGAGAACTATACGTGCCAGATTACTTTTTTG-3’
**Sh-Linc00668-3-antisense**		5'-AGCTCAAAAAAGTAATCTGGCACGTATAGTTCTCTCTTGAAGAACTATACGTGCCAGATTAC-3’
