# Supplementary material for: Sodium new houttuyfonate suppresses metastasis in NSCLC cells through the Linc00668/miR-147a/slug axis
Source: J Exp Clin Cancer Res. 2019 Apr 11;38:155. doi: 10.1186/s13046-019-1152-9 (PMC6458838; doi:10.1186/s13046-019-1152-9)
Supplement: Supplementary file 2 — Table S1. Primers for qRT-PCR. (PDF 36 kb) [file 13046_2019_1152_MOESM2_ESM.pdf]

Table S2. Primers for qRT-PCR

Gene	Primers for qRT-PCR
**Linc00668-F**	GGGTCCAAGGGATCTGCAAG
**Linc00668-R**	CCGCCCAAGATTCCTCTAGC
**E-cadherin-F**	GCTCTTCCAGGAACCTCTGTGATG
**E-cadherin-R**	AAGCGATGGCGGCATTGTAGG
**N-cadherin-F**	GCTCTTCCAGGAACCTCTGTGATG
**N-cadherin-R**	AAGCGATGGCGGCATTGTAGG
**Vimentin-F**	TTGCCGTTGAAGCTGCTAACTACC
**Vimentin-R**	AATCCTGCTCTCCTCGCCTTCC
**Slug-F**	TCAACAGCCAACAAATACCAAG
**Slug-R**	TTCTCCTTGGATTCCTCGTAAG
**GAPDH-F**	CACCCACTCCTCCACCTTTG
**GAPDH-R**	CCACCACCCTGTTGCTGTAG
**miR-147a RT primer**	GTCGTATCCAGTGCAGGGTCCGAGGTATTCGCACTGGATACGACGCAGAA
**miR-147a-F**	CGCGGTGTGTGGAAATGC
**miR-147a-R**	AGTGCAGGGTCCGAGGTATT
**U6 RT primer**	GTCGTATCCAGTGCAGGGTCCGAGGTATTCGCACTGGATACGACAAAATA
**U6-F**	AGAGAAGATTAGCATGGCCCCTG
**U6-R**	ATCCAGTGCAGGGTCCGAGG
