# Supplementary figures and images for: Sodium new houttuyfonate suppresses metastasis in NSCLC cells through the Linc00668/miR-147a/slug axis
Source: J Exp Clin Cancer Res. 2019 Apr 11;38:155. doi: 10.1186/s13046-019-1152-9 (PMC6458838; doi:10.1186/s13046-019-1152-9)

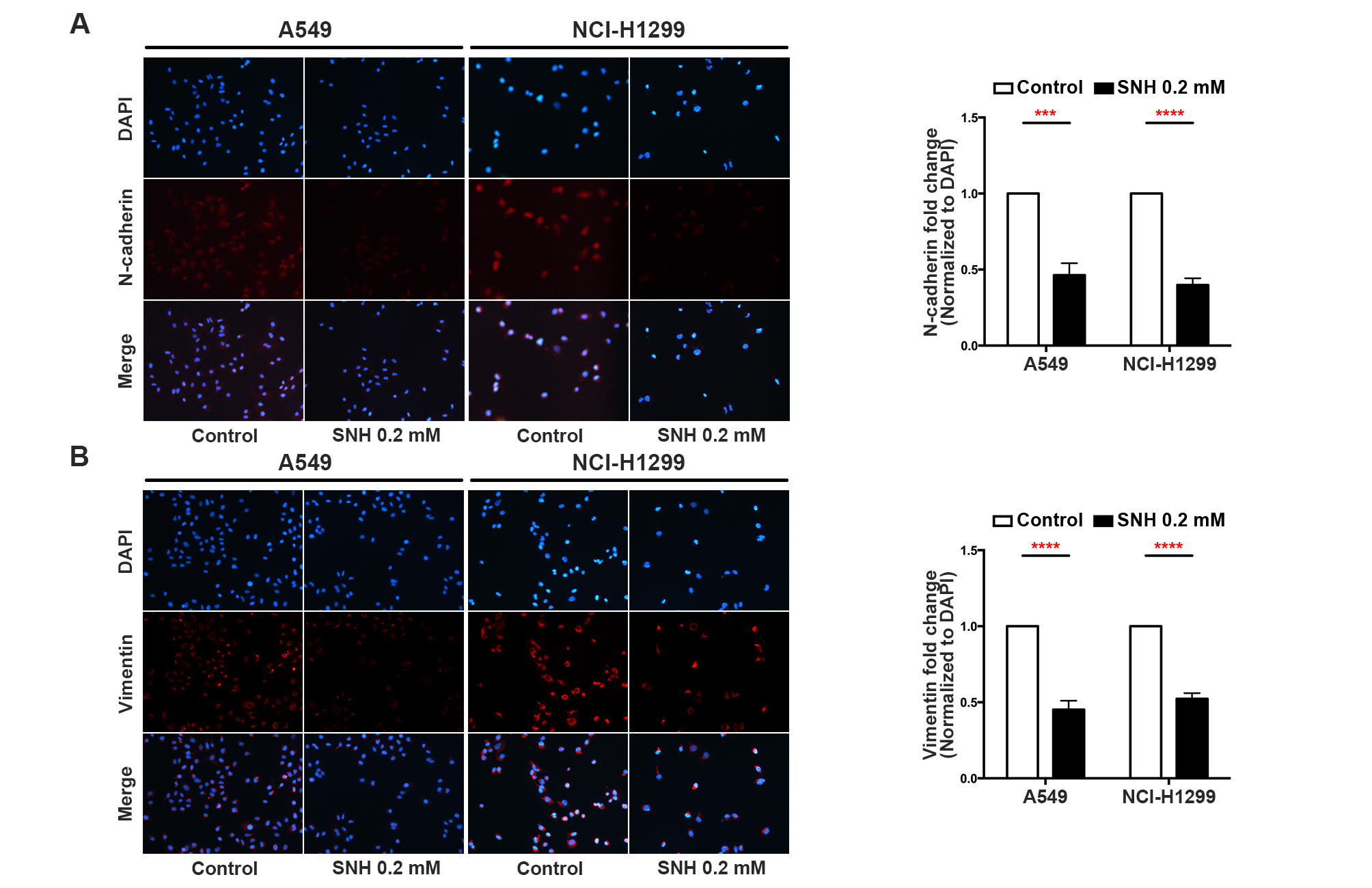

Supplement: Supplementary file 3 — Figure S1 A-B. Immunofluorescence staining of N-cadherin (A) and Vimentin (B) expression in SNH-treated A549 and NCI-H1299 cells. The bars and error bars indicate the mean ± SD. *p < 0.05, **p < 0.01, ***p < 0.005, ****p < 0.001. (TIF 1632 kb) [file 13046_2019_1152_MOESM3_ESM.tif]

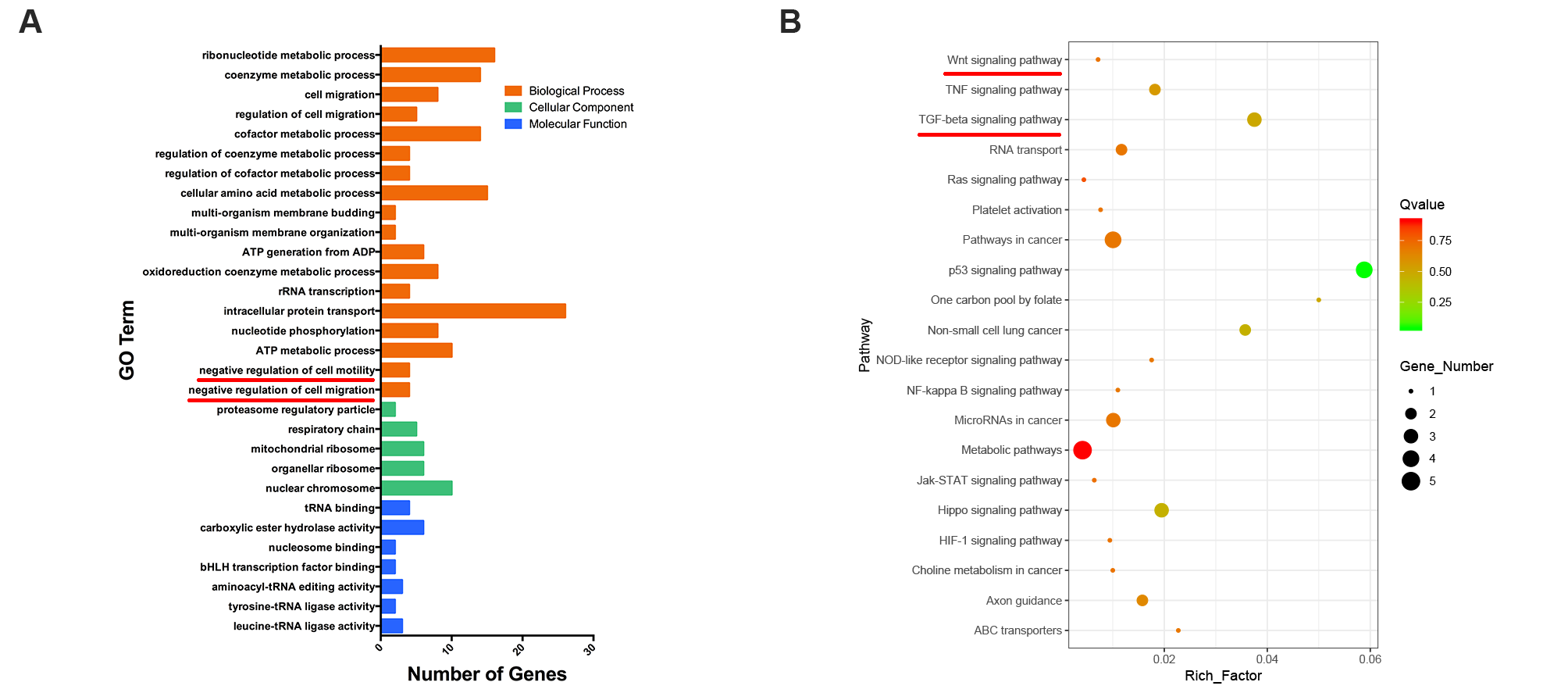

Supplement: Supplementary file 4 — Figure S2 A. Transcriptome analysis, which was based on GO terms, of the screened lncRNA and mRNA sequences in NCI-H1299 cells treated or not treated with SNH for 24 H. B. Pathway analysis, which was based on the KEGG pathway database, of the screened lncRNA and mRNA sequences in NCI-H1299 cells treated or not treated with SNH for 24 H. (TIF 373 kb) [file 13046_2019_1152_MOESM4_ESM.tif]

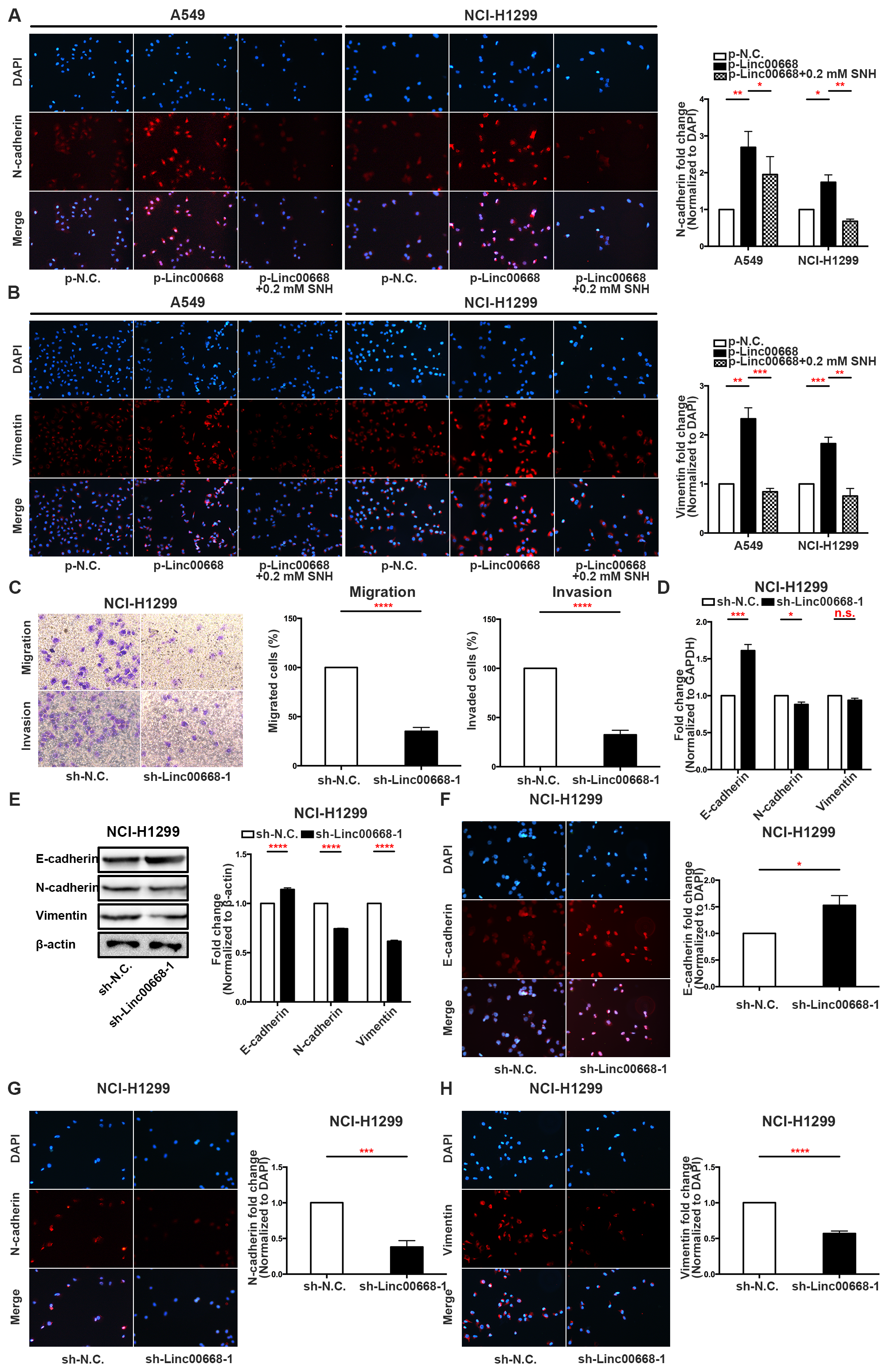

Supplement: Supplementary file 5 — Figure S3,. A-B. Immunofluorescence staining of N-cadherin (A) and Vimentin (B) expression in SNH-treated A549 and NCI-H1299 cells after p-Linc00668 transfection. C. Transwell invasion/migration assays showed that the metastatic ability of NCI-H1299 cells was increased after sh-Linc00668–1 transfection. D. mRNA levels of EMT markers in sh-Linc00668–1-transfected NCI-H1299 cells as determined by qRT-PCR. E. Expression of EMT markers in sh-Linc00668–1-transfected NCI-H1299 cells as determined by western blot analysis. F-H. Immunofluorescence staining of E-cadherin (F), N-cadherin (G) and Vimentin (H) expression in sh-Linc00668–1-transfected NCI-H1299 cells. The bars and error bars indicate the mean ± SD. *p < 0.05, **p < 0.01, ***p < 0.005, ****p < 0.001. (TIF 4296 kb) [file 13046_2019_1152_MOESM5_ESM.tif]

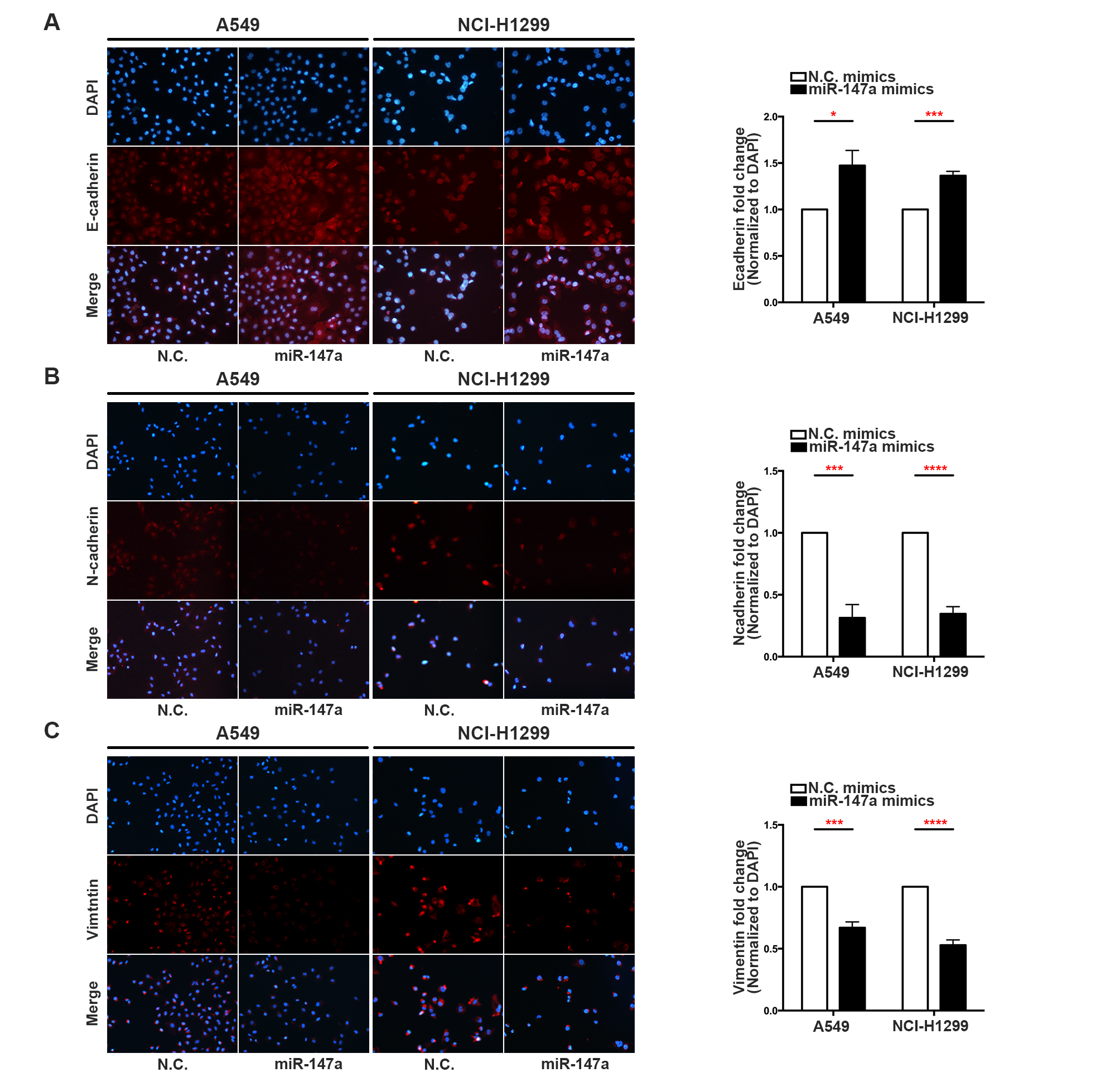

Supplement: Supplementary file 6 — Figure S4. A-C. Immunofluorescence staining of E-cadherin (A), N-cadherin (B) and Vimentin (C) expression in miR-147a mimic-transfected A549 and NCI-H1299 cells. The bars and error bars indicate the mean ± SD. *p < 0.05, **p < 0.01, ***p < 0.005, ****p < 0.001. (TIF 2341 kb) [file 13046_2019_1152_MOESM6_ESM.tif]

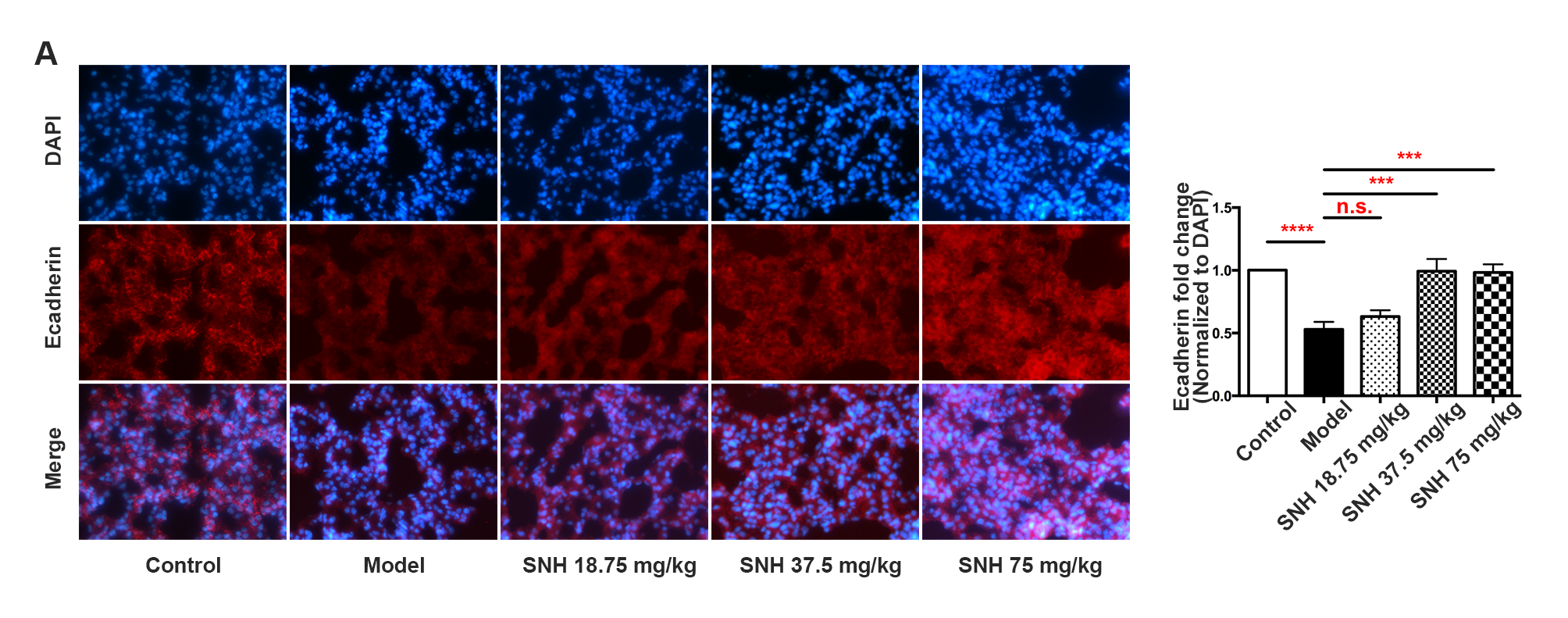

Supplement: Supplementary file 8 — Figure S5. A. Immunofluorescence staining of frozen sections showing E-cadherin expression in the different groups. The bars and error bars indicate the mean ± SD. *p < 0.05, **p < 0.01, ***p < 0.005, ****p < 0.001. (TIF 4677 kb) [file 13046_2019_1152_MOESM8_ESM.tif]
