# Supplementary material for: Sodium new houttuyfonate suppresses metastasis in NSCLC cells through the Linc00668/miR-147a/slug axis
Source: J Exp Clin Cancer Res. 2019 Apr 11;38:155. doi: 10.1186/s13046-019-1152-9 (PMC6458838; doi:10.1186/s13046-019-1152-9)
Supplement: Supplementary file 7 — Table S3. Dual-luciferase report assay vectors. (PDF 34 kb) [file 13046_2019_1152_MOESM7_ESM.pdf]

Table S3. Dual-luciferase report assay vectors

Gene	Vector sequences
**Linc00668-WT**	5'AGGUGACAUCACAUUUGACAGAGGAAGUUACAAAGGUUAUC<br>AACGCCUCGUCUAGUCUAUUCCCAAGAAGGACAGACAACGAAG<br>UUCAAAGUAAGAGAGAAAUUUGUUAUGUUCCAACCGAAGGUA<br>CCAAGGAAGAAUUUCUUACAACUUCCACACAGAAGUCUAAGUA<br>AAUCACAAGCACCUUGGGGUCCCUUUCGACUACAUUUUUGGAG<br>AAAAAAGAGGGUAUACAGAGUUUUUCAACAUAAAAGACCCAG<br>GUUCCCUAGACGUUCGGAGGAUUUCCGUAAAGGUAACAGUGAU<br>GGUGGUCCACACU3'
**Linc00668-MUT**	5'AGGUGACAUCACAUUUGACAGAGGAAGUUACAAAGGUUAUC<br>AACGCCUCGUCUAGUCUAUUCCCAAGAAGGACAGACAACGAAG<br>UUCAAAGUAAGAGAGAAAUUUGUUAUGUUCCAACCGAAGGUA<br>CCAAGGAAGAAUUUCUUACAACUUGGUGUGUGAAGUCUAAGU<br>AAAUCACAAGCACCUUGGGGUCCCUUUCGACUACAUUUUUGGA<br>GAAAAAAGAGGGUAUACAGAGUUUUUCAACAUAAAAGACCCA<br>GGUUCCCUAGACGUUCGGAGGAUUUCCGUAAAGGUAACAGUGA<br>UGGUGGUCCACACU3'
**Slug-WT**	5'CAATTTATGCAATAAGACCTATTCAACTTTTTCTGGGCTGGCCA<br>AACATAAGCAGCTGCACTGCGATGCCCAGTCTAGAAAATCTTTC<br>AGCTGTAAATACTGTGACAAGGAATATGTGAGCCTGGGCGCCCT<br>GAAGATGCATATTCGGACCCACACATTACCTTGTGTTTGCAAGAT<br>CTGCGGCAAGGCGTTTTCCAGACCCTGGTTGCTTCAAGGACACAT<br>TAGAACTCACACGGGGGAGAAGCCTTTTTCTTGCCCTCACTGCAA<br>CAGAGCATTTGCAGACAGGTCAAATCTGAGGGCTCATCTG3'
**Slug-MUT**	5'CAATTTATGCAATAAGACCTATTCAACTTTTTCTGGGCTGGCCA<br>AACATAAGCAGCTGCACTGCGATGCCCAGTCTAGAAAATCTTTC<br>AGCTGTAAATACTGTGACAAGGAATATGTGAGCCTGGGCGCCCT<br>GAAGATGCATATTCGGACGGUGUGUTTACCTTGTGTTTGCAAGA<br>TCTGCGGCAAGGCGTTTTCCAGACCCTGGTTGCTTCAAGGACACA<br>TTAGAACTCACACGGGGGAGAAGCCTTTTTCTTGCCCTCACTGCA<br>ACAGAGCATTTGCAGACAGGTCAAATCTGAGGGCTCATCTG3'
